# Triterpenoids from Chios Mastiha Resin Against MASLD—A Molecular Docking Survey

**DOI:** 10.3390/cimb47010051

**Published:** 2025-01-15

**Authors:** Nataša Milošević, Maja Milanović, Milica Medić Stojanoska, Varomyalin Tipmanee, Ilias Smyrnioudis, George V. Dedoussis, Nataša Milić

**Affiliations:** 1 Department of Pharmacy, Faculty of Medicine, University of Novi Sad, 21000 Novi Sad, Serbia; natasa.milosevic@mf.uns.ac.rs (N.M.); natasa.milic@mf.uns.ac.rs (N.M.); 2 Faculty of Medicine, University of Novi Sad, 21000 Novi Sad, Serbia; milica.medic-stojanoska@mf.uns.ac.rs; 3 University Clinical Center of Vojvodina, 21000 Novi Sad, Serbia; 4 Department of Biomedical Sciences and Biomedical Engineering, Faculty of Medicine, Prince of Songkla University, Songkhla 90110, Thailand; tvaromya@medicine.psu.ac.th; 5 Chios Mastic Gum Growers Association, 82150 Chios, Greece; ismyrnioudis@gummastic.gr; 6 Department of Nutrition and Dietetics, School of Health Science and Education, Harokopio University of Athens, 17671 Athens, Greece; dedousi@hua.gr

**Keywords:** non-alcoholic liver disease, natural compounds, biological activity, antiobesity, molecular docking, molecular target

## Abstract

Non-alcoholic fatty liver disease (NAFLD) is the most common chronic liver disease without an approved pharmacological approach for its prevention/treatment. Based on the modified Delphi process, NAFLD was redefined as metabolic dysfunction-associated steatotic liver disease (MASLD) to highlight the metabolic aspect of liver pathogenesis. Chios mastiha (*Pistacia lentiscus* var. *Chia*, Anacardiaceae) resin demonstrated promising results in MASLD treatment. In this paper, molecular docking was applied to test 16 compounds from Chios mastiha as potential ligands for the receptors GR, LXRα, LXRβ, PPARα PPARγ, MC4R, AMPK, and VEGFR2, whose up- and down-regulation interfere with MASLD development and progression. The observed compounds had moderate and high affinity for LXR, GR, MC4R, and PPARγ in comparison to proven ligands, while their affinity for PPARα, AMPK, and VEGFR was less pronounced. The combination of active compounds from Chios mastiha rather than a single molecule may have a superior ability to control the intertwined MASLD metabolic pathways.

## 1. Introduction

Non-alcoholic fatty liver disease (NAFLD) is a multisystem disease that affects around 25% of adults worldwide. As a hepatic component of metabolic syndrome, NAFLD is characterized by liver steatosis in more than 5% of hepatocytes with metabolic risk factors, especially obesity and type 2 diabetes mellitus (T2DM). When diagnosing NAFLD, excessive alcohol consumption (≥30 g/day for men and ≥20 g/day for women) should be excluded [1]. In 2023, a modified Delphi process led by three large pan-national liver associations resulted in a new nomenclature for fatty liver disease, namely, metabolic dysfunction-associated steatotic liver disease (MASLD). The criteria for MASLD diagnosis require the concomitant presence of hepatic steatosis and at least one of the five cardiometabolic risk factors: increased waist circumference, arterial hypertension, hypertriglyceridemia, low high-density cholesterol (HDL-C), prediabetes, or insulin resistance [2]. Inflammation and angiogenesis are two pivotal processes that may occur simultaneously with fat accumulation and contribute to disease progression. The infiltration of neutrophils and T-lymphocytes into portal tracts correlates with disease severity, and the production of IL-17 contributes to liver injury [3]. Furthermore, inflammation and hypoxia trigger angiogenesis, which promotes fibrosis progression, induces enhanced lipid absorption, and exacerbates the disease. The production of new blood vessels is not only a quantitative process but also an alternative qualitative process in chronic liver disease, where liver sinusoidal endothelial cells lack their fenestrae [4]. The therapeutic approach for MASLD, therefore, requires multitarget pharmacological agents that will disrupt liver fat accumulation, inhibit inflammation, and disrupt pathological angiogenesis without dysregulating glucose and lipid homeostasis and compromising the immune system’s protective functions.

Chios mastiha (*Pistacia lentiscus* var. *Chia*, Anacardiaceae) resin is a unique gum derived from mastic trees cultivated in the southern part of Chios, a Greek island in the northern Aegean Sea. It has been recognized as a Protected Designation of Origin (PDO) product by the European Union [5]. Chios mastic gum has been used to treat gastrointestinal disorders and for the production of cosmetics for centuries, and it has been recently rediscovered as a natural product with valuable pharmacological potential, including antibacterial, anti-inflammatory, antioxidant, antidiabetic, cardioprotective/anti-atherogenic, gastroprotective/anti-ulcer, and anti-cancer properties. The European Medicines Agency (EMA) recognizes the therapeutic effects of Chios mastic resin in mild dyspeptic disorders and in treating skin inflammation and healing minor wounds [6]. The pharmacological activity of mastic gum is attributed to over 120 identified chemical compounds in the resin. The primary group of active compounds in Chios mastiha are triterpenoids, which constitute up to 70% of the resin [7,8]. About 25% of the resin consists of an inactive and insoluble poly-β-myrcene polymer, which binds with triterpenes, thus limiting their activity. The poly-β-myrcene polymer is precipitated through specific time-consuming and resource-intensive processes to isolate the active compounds. Therefore, the full potential of Chios mastiha triterpenoids remains unclear [8]. The natural mixture of chemically different compounds with synergistic pharmacological activity found in Chios mastiha resin may offer an ideal combination of molecules that target various pathological features of MASLD.

Molecular docking is a computational tool that allows the screening of the ligand-based activation of specific receptors that control biological processes [9,10]. Ligand binding to a protein triggers interactions that activate or inhibit certain metabolic pathways, making it the foundation for rational drug design strategies. This can be achieved by using the publicly available and experimentally determined structure of proteins, nucleic acids, and their complexes with ligands [11,12,13,14]. Despite their limitations, computer-based simulations are highly efficient and result in significant time and resource savings. The rapid development of computer-based tools has led to the development of an all-computational drug design protocol [15].

Considering the efficacy of Chios mastiha resin in NAFLD [16,17,18], this paper applies molecular docking, which is a powerful tool, to examine the affinity of 16 compounds isolated from the resin for designated receptors. The receptors analyzed in this study were carefully selected after reviewing various pathways in MASLD development and progression and have already been reported as potential targets in NAFLD therapy [19]. For the first time, individual molecules from the Chios mastiha resin are examined as ligands for receptors whose up- or down-regulation defines the outcomes of MASLD.

## 2. Materials and Methods

### 2.1. Overview of Important Pathways and Factors That Contribute to MASLD

NAFLD, as a hepatic manifestation of combined metabolic disorders, represents a significant burden on healthcare systems globally, while therapeutic approaches remain largely unaddressed. The shift from NAFLD to MASLD emphasizes the metabolic aspect of liver disease. A variety of interconnected molecular pathways initiate lipid accumulation in the liver, which progressively exacerbates into an inflammatory state and may eventually lead to hepatocellular carcinoma or liver cirrhosis [20,21].

Obesity is a key metabolic driver of NAFLD; therefore, the rising global prevalence of obesity is followed by an increase in NAFLD cases [22]. Among lean NAFLD patients, a more disrupted metabolic profile has been observed with a higher prevalence of hypertension, insulin resistance, dyslipidemia, and metabolic syndrome, all accompanied by elevated inflammatory markers [23]. T2DM appears to be the single most important risk factor for the accelerated progression of MASLD. Advanced fibrosis and more severe liver histology were observed in diabetic patients with NAFLD [24]. Insulin resistance is considered the primary pathway in NAFLD and a pivotal risk factor for fibrosis progression [25]. Another mechanism for NAFLD development includes oxidative stress with lipid peroxidation and mitochondrial dysfunction [26]. Therefore, targeting different metabolic pathways should lead to the optimization of MASLD/MASH treatment.

Peroxisome proliferator-activated receptors (PPARs) are recognized as lipid and glucose metabolism regulators and as targets for therapeutic intervention in type 2 diabetes mellitus and metabolic syndrome. PPARα activation is associated with accelerated lipolysis, fatty acid formation, and enhanced apolipoprotein expression, which results in increased high-density lipoprotein cholesterol (HDL-C) plasma levels and reduced low-density lipoprotein cholesterol (LDL-C) levels [27]. Thus, PPARα agonists are considered effective in improving dyslipidemia [28]. In addition, PPARα regulates the lipogenic pathways in the liver both directly and indirectly. PPARα agonists enhance human sterol regulatory element binding protein-1c (SREBP-1c) transcription by directly interacting with the human SREBP-1c promoter, and they also indirectly modulate SREBP-1c transcriptional activity through the LXR signaling pathway [29]. SREBP-1c is an insulin-dependent transcription factor involved in glucose utilization and fatty acid synthesis. Furthermore, it has been reported that SREBP-1c promotes hepatic steatosis in humans diagnosed with hyperhomocysteinaemia or in those who consume an excessive amount of alcohol. In patients with diabetes mellitus who have reduced insulin levels and episodes of hyperglycemia, SREBP-1c is also activated through endoplasmic reticulum (ER) stress, leading to lipid synthesis and lipotoxicity [30]. PPARα acts as an insulin antagonist in glucose and lipid homeostasis by decreasing glycolysis, stimulating glycogen synthesis, and inhibiting lipid accumulation. PPARα agonism reduces intrahepatic lipid accumulation, which in turn inhibits liver inflammation and fibrosis and normalizes histological changes in hepatocytes [31]. The positive effects of PPARα agonists include enhanced hepatic lipid metabolism due to stimulated CYP4A-mediated ω-oxidation, as well as peroxisomal and mitochondrial β-oxidation. PPARα upregulation also reduces the number of activated macrophages and stellate cells in the liver, along with fibrotic markers [29]. The beneficial effects of mastiha resin on non-alcoholic steatohepatitis (NASH) mediated by PPARα were confirmed in an animal model. Mice on a high-fat diet showed lower body weight and total cholesterol and LDL levels than the control group and did not develop insulin resistance. This effect was completely absent in PPARα−/−mice [32]. There are contrasting metabolic effects between activated PPARα and PPARγ as PPARγ improves insulin resistance. PPARγ agonism results in decreased free fatty acid content in all organs except adipose tissue and circulating blood. It enhances insulin effects by increasing lipid transfer from the circulation, the liver, and skeletal muscles into white adipose tissue, which stimulates adipose tissue to store triglycerides through upregulated adipogenesis. PPARγ also induces white adipose tissue to secrete adiponectin and leptin. Adiponectin promotes hepatic glucose output, while leptin regulates the feeding behavior [27]. Fibrates, i.e., PPARα agonists, have been used as a therapeutic tool for hypertriglyceridemia and hypercholesterolemia while thiazolidinediones, i.e., PPARγ agonists, have been used for hyperglycemia treatment. Research programs have been undertaken to develop both PPARα- and PPARγ-selective agonists that combine their therapeutic effects, with high expectations in the treatment of type 2 diabetes mellitus and metabolic syndrome [28]. Dual-PPARα/γ agonists (glitazars) have demonstrated their beneficial metabolic effects but, paradoxically, have led to aggravated congestive heart failure in type 2 diabetes mellitus patients [33].

The effects of glucocorticoids (GCs) are mediated through the glucocorticoid receptor (GR) and include the regulation of metabolic pathways in the liver. GR activation enhances the influx of free fatty acids and lipoproteins, increases de novo lipogenesis, and promotes lipid efflux through very-low-density lipoprotein (VLDL) synthesis. The mechanism behind glucocorticoid action in liver impairment is multilayered and complex as GCs interfere with metabolic pathways that both induce and prevent steatosis. Glucocorticoid receptors on the hepatocytes play a pivotal role in managing direct and indirect transcriptional regulations that promote insulin resistance, hyperglycemia, and fatty liver [34]. Lipid accumulation in the liver due to GR expression is a precursor for hepatic inflammation, which promotes the progression of MASLD. Conversely, excessive GC exposure and GR antagonism both lead to liver steatosis and fibrosis [35].

Activated protein kinase (AMPK) has also been identified as a target in the therapeutic approach for MASLD. Inflammation, obesity, and diabetes mellitus reduce AMPK activity. AMPK agonism may be beneficial in MASLD therapy through three possible mechanisms: inhibition of de novo lipogenesis, promotion of fatty acid oxidation, and maintenance of the mitochondrial function in adipose tissue. AMPK agonists may be considered as therapeutic agents against MASLD/MASH only if their activity is time-limited as chronic AMPK activation can lead to obesity and TG deposition in the liver [36]. Nevertheless, in vivo, AMPK activation with doxycycline reduced lipid levels through a dual mechanism: enhanced fatty acid oxidation and limited de novo lipogenesis. Induced AMPK activity in the liver also led to hypertriglyceridemia and hypercholesterolemia [37]. In addition to the effects of liver-specific AMPK activation, stimulated AMPK activity prevented lipid accumulation in the liver [38]. The AMPK activator PF-06409577 not only reduced lipid accumulation in the liver but also decreased hepatic inflammation and fibrosis markers in a developed NASH model. It was presumed that AMPK activation inhibited SREBP-1 transcriptional activity, leading to a decline in lipogenic gene expression [39]. Metformin, an insulin sensitizer, is also recognized as an AMPK activator. Studies conducted on diabetic NAFLD patients treated with metformin for 12 to 24 weeks resulted in reductions in body mass index, liver fat content, and liver enzymes, along with improved insulin resistance. Although the importance of AMPK activation with metformin was confirmed in clinical settings, no improvement in liver fibrosis was observed among diabetic patients with confirmed NASH, who were treated with metformin [40], suggesting that a single therapy approach is insufficient.

Neurons in the hypothalamic arcuate nucleus are affected by metabolic hormones, including leptin, insulin, ghrelin, and nutrients, which regulate the expression of orexigenic or anorexigenic neuropeptides like proopiomelanocortin (POMC). POMC releases α-melanocyte-stimulating hormone (α-MSH), which binds with melanocortin-4 receptors (MC4Rs). Since food intake and energy expenditure are controlled by MC4Rs, mutations in the MC4R gene cause hereditary obesity in humans. Additionally, the loss of MC4R function has been observed to result in insulin resistance and dyslipidemia. In vivo, MC4R deletion induces hyperphagia, followed by obesity [41]. MC4R agonists are recognized as a potential therapeutic tool for MASLD and MASH. Recent studies using daisaikoto, a combination of various crude drugs, showed an improvement in liver enzymes and total cholesterol and total protein levels compared to the control group. Moreover, daisaikoto recovered heat production and basal metabolism inhibited by MC4R deficiency [42].

Finally, nuclear receptors are also potential targets for MASH therapy. Liver X receptor (LXR) exhibits surprisingly contradictory metabolic effects that both induce and inhibit MASLD progression. LXR activation not only leads to cholesterol homeostasis modulation, induces anti-inflammatory effects, and promotes increased insulin sensitivity but also triggers liver lipid accumulation and produces hypertriglyceridemia. On the other hand, liver LXR antagonism can be desirable in MASLD treatment as it alleviates hepatic steatosis, inflammation, and fibrosis, but it increases the risk for cardiovascular adverse effects [43]. The LXR agonist T0901317 elevated insulin sensitivity by upregulating glucose receptor GLUT4 expression in white adipose tissue in an experiment [44], while in vivo, T0901317 increased the blood glucose concentration and, paradoxically, downregulated glucose uptake [45]. Another LXR agonist, GW3965, influenced the glucose metabolic pathway and improved glucose tolerance [46], but both T0901317 and GW3965 elevated plasma and liver triglyceride levels [47]. Interestingly, oltipraz, a LXRα inhibitor, applied to NAFLD patients in a double-blind and placebo-controlled study led to decreased liver fat content and elevated total cholesterol compared to the control [48]. LXR is recognized as an inhibitor of pro-inflammatory gene activation, thus modulating the inflammatory response in conditions such as atherosclerosis, lupus, dermatitis, neuroinflammation, and arthritis. Additionally, LXR antagonists reduced cytokine production [49] and indirectly exhibited anti-inflammatory action in the liver, likely by regulating cholesterol and phospholipid metabolism. The LXR antagonist, SR9238, reduced liver collagen deposition and ameliorated liver fibrosis [50]. LXR is overexpressed in NAFLD patients, with increased activity corresponding to NASH progression. LXR directly controls the activity of SREBP-1c, and LXR deletion limits the effects of SREBP-1c, which decreases lipogenesis. Interestingly, natural compounds have been reported to alleviate NAFLD by both activating and inhibiting LXR [43].

Angiogenesis contributes to the progression of MASLD. Serum levels of angiogenic markers such as VEGF and soluble VEGFR-1 (sVEGFR1) and sVEGFR2 in NASH and/or NAFLD patients were elevated compared to controls; VEGFR overexpression was also observed [51]. It has also been reported that proangiogenic genes are activated in the early stage of NAFLD development before the disease progresses to NASH. Steatosis and hypoxia provoke changes in vascular architecture. Steatosis itself produces hypoxia in the liver by mechanically disturbing oxygen transport, increasing pressure on the liver sinusoids, and enhancing oxygen consumption due to elevated fatty acid metabolism. In addition, leptin stimulates angiogenesis [4]. Preventive and therapeutic applications of anti-VEGFR2 agents reduced steatosis and inflammation [52]. The effects of vascular endothelial growth factor-C (VEGF-C) are not limited to angiogenesis and lymphangiogenesis; surprisingly, it also acts as a potential regulator of cholesterol metabolism by maintaining lymphatic vessels and, consequently, regulating fat absorption. The overexpression of VEGF-C leads to increased weight gain and lipid accumulation [53]. Targeting VEGF represents a meaningful intervention in MASLD treatment.

Enhanced fatty acid metabolism in NAFLD patients, which includes peroxisomal oxidation, microsomal oxidation, and ER ω-oxidation, inevitably generates ROS in hepatocytes. A reduction in antioxidative capacity leads to oxidative stress, resulting in lipid peroxidation, damage to plasma membrane integrity, and cell death. Lipid peroxidation yields 4-hydroxynonenal (4-HNE) and malondialdehyde (MDA), which trigger inflammation events. Inflamed adipose tissue macrophages secrete pro-inflammatory cytokines that exacerbate inflammation [54,55].

### 2.2. Analyzed Compounds

**Figure 1:**
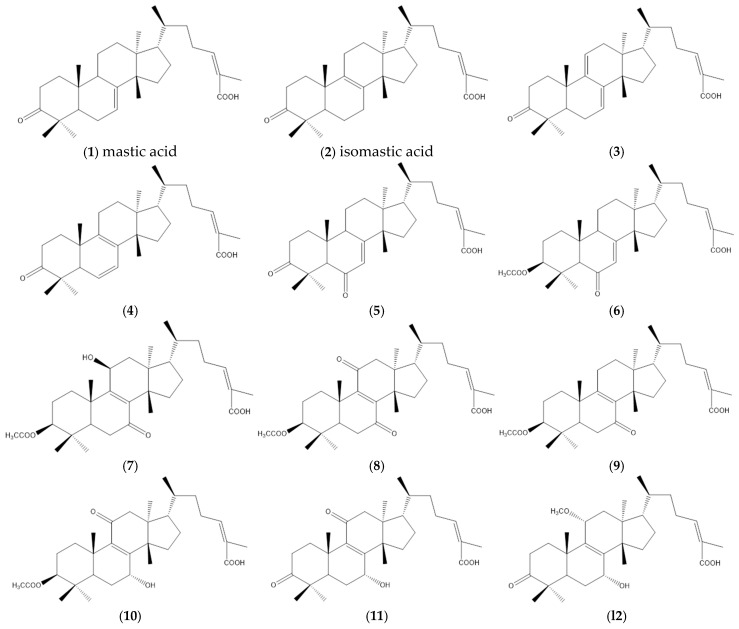

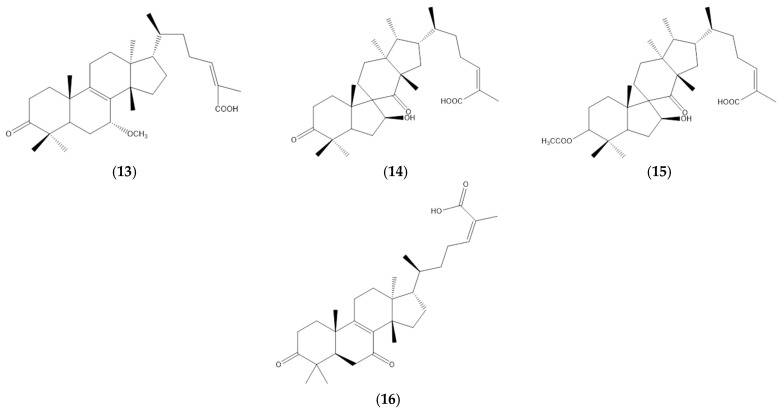
Structures of the analyzed triterpenoids (**1**–**16**) isolated from Pistacia lentiscus.

In this paper, 16 triterpenoids (Figure 1) isolated from *Pistacia lentiscus* were analyzed. High-resolution mass spectroscopy was applied to confirm compounds **1** and **2** [56], while compounds **3**–**16** were confirmed through 1H NMR and 13C NMR analyses [57]. ChemDraw Professional (version 16.0) was used to draw their 2D structures and generate Simplified Molecular-Input Line-Entry System (SMILES) specifications.

All examined 16 compounds have the following properties: molecular mass < 550 g/mol (and molecular volume < 550 Å^3^), hydrogen bond acceptors ≤ 6, hydrogen bond donors ≤ 2, polar surface area ≤ 100 Å^2^, lipophilicity (logP ≤ 6.5), and solubility (logS ≥ −6.85).

### 2.3. Molecular Docking

Genetic Optimisation for Ligand Docking (GOLD) version 2020.3.0 was applied for molecular docking of the analyzed triterpenoids toward the following receptors: GR, LXRα, LXRβ, PPARα, PPARγ, MC4R, AMPK, and VEGFR2. The 3D structures of the 16 observed compounds were optimized to the most energetically favorable conformations using Chem3D (version 16.0). The three-dimensional crystal structures of the proteins are available in pdb format from the RCSB Protein Data Bank (https://www.rcsb.org/, accessed on 20 July 2024). The triterpenoids docking analysis toward PPARα, PPARγ, GR, and MC4R receptors was conducted on proteins with proven ligands: GW (PDB code 2P54 for PPARα) [58], amorfrutin 1 (PDB code 2YFE for PPARγ) [59], deacylcortivazol (PDB code 3BQD for glucocorticoid receptor) [60], and SHU9119 (PDB code 6W25 for melanocortin-4 receptor) [61]. All the aforementioned compounds act as agonists to the observed receptors, except SHU9119. In addition, docking analysis was conducted for LXRα and LXRβ on proteins with the following confirmed agonists: benzisoxazole (PDB code 3IPS for liver X receptor α and 24(S),25-epoxycholesterol (PDB code 1P8D for liver X receptor β) [62,63]. For AMPK, docking analysis was performed on the protein (PDB code 4CFF) with the recognized agonist STU1552 [64]. Finally, for VEGFR2, molecular docking was conducted on the protein (PDB code 4ASD) with sorafenib as the confirmed inhibitor of VEGFR2 activity [65].

The water molecules were removed from the protein structures, and binding sites on each protein were defined in a radius of 6 Å of the co-crystalized ligand to provide the RMSD value in the reliable interval of 2 Å. The ChemPLP function was used to determine the most optimal energetic space orientation of the analyzed ligand and its interaction with target binding sites. As a result, 10 of the most favorable conformations were generated for each analyzed compound and target and were ranked according to their score [66]. The highest value obtained was only used for further analysis because it indicates the best ligand-target pose [67,68]. All sixteen analyzed compounds underwent molecular docking for each of the abovementioned receptors, respectively, in order to obtain their ChemPLP fitness score [69]. Maestro academic version 13.8 was used to visualize the docking results.

## 3. Results

The ChemPLP score values for the selected receptors (PPARα, PPARγ, GR, and MC4R) are presented in Table 1, comparing all 16 analyzed compounds to the proven ligands.

**Figure 2:**
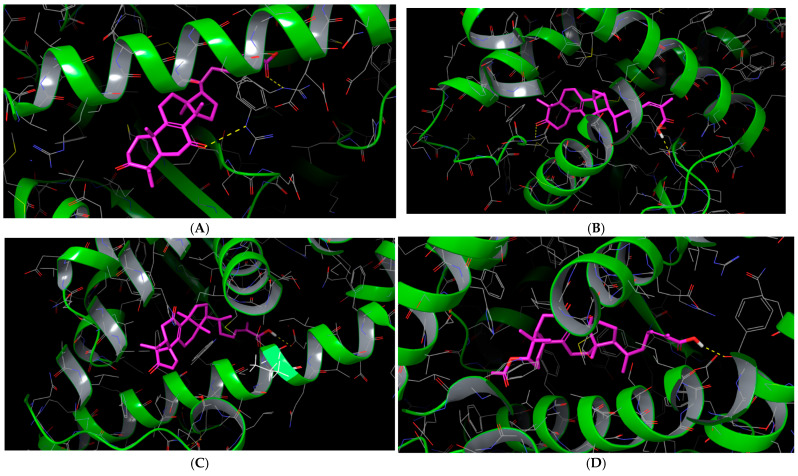
The best binding conformations between (**A**) compound 16 and PPARγ receptor; (**B**) compound 2 and GR; (**C**) compound 16 and LXRα; and (**D**) compound 9 and LXRβ based on ChemPLP scores. Compound is presented in purple colour while protein chains in green. The yellow dashed lines indicate hydrogen bonds between compound and involved amino acid residues.

In comparison to the proven ligand GW, the observed triterpenoids exhibited less pronounced affinity toward the PPARα receptor (2P54). Compounds **6**, **7**, **9**, and **16** demonstrated the highest affinity toward the PPARα receptor (Table 1). The affinity of the analyzed compounds toward the PPARγ receptor (2YFE) was also less pronounced compared to the known ligand amorfrutin **1**. However, compounds **2**, **3**, **4**, and **16** (Table 1) expressed the most potential to act as ligands. For compound 16, the highest affinity was observed towards the PPARα receptor (2P54) and the PPARγ receptor (2YFE), as shown in Figure 2A. Compounds **2**, **3**, and **4** were also the most compatible as ligands for GR (3BQD), although all the observed molecules had a lower capacity to bind with GR compared to the proven ligand, i.e., deacylcortivazol (Table 1). The binding conformations between compound **2** and GR (3BQD) are shown in Figure 2B. The investigated molecules had a high potential to bind with MC4R (6W25), with compounds **5**, **6**, **7**, **8**, **9**, **10**, **15**, and **16** exhibiting even higher affinity than SHU9119 (Table 1).

The binding affinity of the investigated compounds quantified by ChemPLP scores toward AMPK in comparison to the recognized ligand STU1552 is summarized in Table 2. Table 3 presents ChemPLP scores for LXRα and LXRβ compared to the validated ligands benzisoxazole and 24(S),25-epoxycholesterol, respectively. Table 4 lists ChemPLP scores for VEGFR2 for all investigated compounds, with the confirmed ligand sorafenib (BAY 43-9006) included for comparison.

The affinity of the analyzed molecules toward AMPK (4CFF) is not as pronounced as the confirmed ligand STU1552 (Table 2). However, compound **16** demonstrated the highest ligand-like behavior toward AMPK. Although all observed compounds had a lower capacity to bind with LXRα (3IPS) compared to the proven ligand benzisoxazole, their ChemPLP scores were still high (Table 3). Once again, compound 16 exhibited the highest compatibility as a ligand for LXRα (Figure 2C). The ability to bind with LXRβ (1P8D) was more pronounced for all observed compounds compared to their affinity toward LXRα. Compound **9** had more potential to bind with LXRβ (Figure 2D), even compared to 24(S),25-epoxycholesterol as the known ligand (Table 3), while the ChemPLP scores of compounds **6**, **7**, and **8** were also considerably high. The affinity of the observed compounds toward VEGFR2 (4ASD) was modest in comparison to sorafenib (BAY 43-9006) as the recognized ligand (Table 4). The highest affinity for binding with VEGFR2 was demonstrated by compounds **1**, **3**, **5**, and **6**.

## 4. Discussion

Chios mastiha resin has been shown to be a compelling therapeutic agent in NAFLD patients, as demonstrated in an open randomized, double-blind, and placebo-controlled study conducted in three centers (Greece, Italy, and Serbia). Lipid metabolite levels decreased in NAFLD patients treated with Chios mastiha resin for six months. In obese NAFLD patients, Chios mastiha resin reduced liver inflammation and fibrosis [16]. The total antioxidant status was higher among patients treated with Chios mastiha resin in comparison to the control [18]. Mastiha demonstrated anti-inflammatory effects in inflammatory bowel disease, particularly in ulcerative colitis, by preventing an increase in plasma miR-155, a mediator in T helper-17 (Th17) differentiation. This effect was less pronounced in patients with NAFLD [17]. The effects of proper diet and weight reduction as a non-pharmacological treatment for MASLD should not be neglected. A “Low-Fat Dairy and Poultry” diet alleviated liver inflammation and fibrosis in NAFLD/NASH patients [70].

Undoubtedly, Chios mastiha resin is beneficial for MASLD/MASH patients, predominantly in ROS scavenging and preventing inflammation and fibrosis. Additionally, Chios mastiha resin may interfere with the complex metabolic pathways involved in MASLD development and progression through various receptors, as demonstrated in this study. The observed compounds from Chios mastiha resin showed high affinity for LXR, GR, MC4R, and PPARγ (Table 1 and Table 3) compared to proven ligands, while their ability to bind to PPARα, AMPK, and VEGFR was less pronounced (Table 1, Table 2 and Table 4). It can be stated that different molecules isolated from Chios mastiha resin have a high binding affinity for each receptor, respectively (Figure 1). The results suggest that a combination of molecules, rather than a single compound from Chios mastiha resin, contributes to its beneficial effects in MASLD patients. It has already been suggested that combined therapeutic intervention for NAFLD may be more efficient than mono-therapy [71]. Although it is typically assumed that a combination of compounds from the same natural product will have additive or synergistic effects, the mixture of molecules from Chios mastiha resin may have antagonistic effects on the same receptors or on different receptors that control the same metabolic pathways. The contrasting activity may be even more desirable as the chronic activation of some receptors can produce adverse effects, exacerbating the disease.

**Figure 3:**
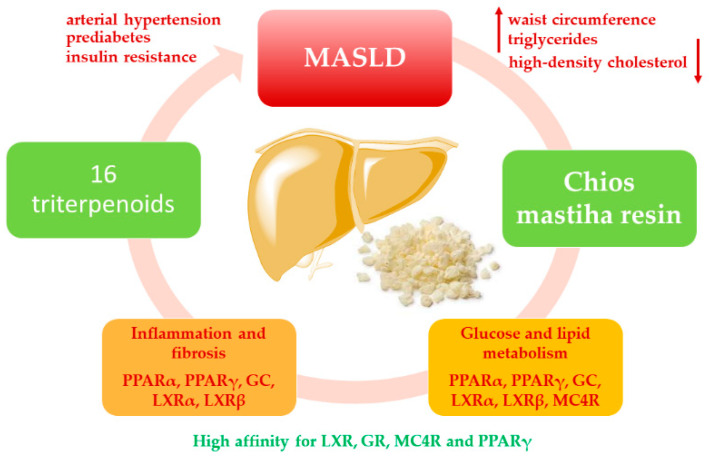
The potential mechanisms of the effects of Chios mastiha resin on MASLD.

Molecular docking evaluates the energies required for a molecule to bind to the ligand site of a defined protein, but whether the bond induces or inhibits the activation of a particular receptor remains unclear. In silico analysis has limited ability to predict whether the ligand–protein complex produces chronic effects or whether it has time-limited activation of a metabolic pathway dependent on the observed receptor. Avoiding the chronic activation of certain targets is another challenge that should be addressed and may also be vital in optimizing MASLD therapies. Despite all limitations, the data presented in this paper can be a starting point for further in vitro/in vivo analysis of the effects of Chios mastiha resin or the activity of a particular molecule isolated from mastiha on a specific receptor that regulates defined metabolic processes (Figure 3). The investigated receptors (Table 1, Table 2, Table 3 and Table 4) control processes that are pivotal not only in MASLD/MASH treatment but also in other diseases, such as atherosclerosis (LXR and PPARα), dyslipidemia (PPARα), type 2 diabetes mellitus (PPARγ, MC4R, and AMPK), obesity (PPARγ, MC4R, and AMPK), metabolic syndrome (GR, LXR, and PPARα), anorexia (MC4R), cardiovascular diseases (PPARγ and AMPK), neuroinflammation (LXR and AMPK), autoimmune diseases (GR), cancer (GR, PPARγ, and AMPK), etc. The potential antioxidant and anti-inflammatory effects of observed compounds could also be applied therapeutically to sarcopenia, which is closely linked to MASLD. In fact, sarcopenia and MASLD share similar pathogenetic features, including insulin resistance, systemic inflammation, and adiponectin dysregulation, that could be alleviated by natural products [72]. Therefore, specific molecules from Chios mastiha resin, or the resin itself, may be considered potential therapeutic agents in all the aforementioned diseases, whose progression may be influenced by the activation or inhibition of the selected receptors (Table 1, Table 2, Table 3 and Table 4). The 16 examined compounds exhibit desirable properties in terms of the physico-chemical characteristics expected from drug candidates. They may be considered both as drug candidates and as precursors for the design of new molecules with an enhanced affinity for specific receptors or improved pharmacokinetic properties.

## 5. Conclusions

The therapeutic strategy for MASLD remains unresolved. Natural products, which contain a combination of active compounds that bind to different receptors, exhibiting both the activation and inhibition of target proteins, could provide a solution to the complex and contradictory effects of key receptors and their pathways in MASLD development and progression. The mixture of various active molecules in herbal extracts, such as Chios mastiha resin, may also be excellent adjuvant therapy in combination with synthetic drugs for MASLD treatment. Considering that binding affinity differs per compound and per observed receptor, based on the obtained docking scoring values, the combination of active compounds from Chios mastiha might be more effective in controlling MASLD pathways than the application of a single triterpenoid. The molecular docking approach was applied as an inexpensive, environmentally friendly, and animal cruelty-free alternative for quick progress in studying the mechanism of action of Chios mastiha compounds. Hence, this study highlights all the benefits and limitations of in silico analysis. Despite the improvements in algorithms and scoring functions, receptor flexibility is still a significant challenge in flexible docking. In addition, the inability to predict the effective concentration of compounds required for the activation of the observed receptors is the main restriction of in silico analysis. The obtained results, in combination with further preclinical studies, could enable a better understanding of MASLD and promote clinical studies that explore new therapeutic approaches for MASLD. Finally, the analyzed compounds may be recognized as forerunners in the development of new molecular entities for the treatment of various metabolic imbalances in which the aforementioned receptors are implicated.

## Figures and Tables

**Table 1:** The binding affinity of the analyzed compounds expressed as ChemPLP scores for PPARα (2P54) in comparison to GW, PPARγ (2YFE) in comparison to amorfrutin 1, GR (3BQD) in comparison to deacycortivazol, and MC4R (6W25) in comparison to SHU9119.

Receptor (Code) Compound	PPARα (2P54)	PPARγ (2YFE)	GR (3BQD)	MC4R (6W25)
GW	98.5022			
amorfrutin 1		95.3068		
deacylcortivazol			122.6605	
SHU9119				69.1986
**1**	54.0050	70.6616	82.8365	67.2488
**2**	52.7068	75.7911	87.8871	66.8322
**3**	54.8065	74.5059	84.3943	68.0783
**4**	52.8967	74.1974	84.8618	67.9963
**5**	53.7275	70.0614	79.9776	70.8568
**6**	56.7780	70.1652	72.0166	76.4401
**7**	56.5781	68.7686	67.6894	73.6959
**8**	54.9620	66.6752	64.5330	73.9571
**9**	56.0697	67.3736	76.6749	71.7798
**10**	50.3439	67.5160	65.7566	70.2063
**11**	50.6076	69.9708	81.2989	64.5848
**12**	34.0140	69.3329	68.9301	64.0505
**13**	51.5451	69.4731	76.4200	67.5745
**14**	40.3146	61.5978	68.2356	68.1883
**15**	50.3570	63.0744	46.5814	71.5788
**16**	56.9901	76.5888	75.2126	69.4968

**Table 2:** The affinity of the investigated compounds quantified with ChemPLP scores for AMPK (4CFF) compared to the recognized ligand STU1552.

Receptor (Code) Compound	AMPK (4CFF)
STU1552	99.7299
**1**	57.3378
**2**	51.6196
**3**	48.5665
**4**	53.1452
**5**	54.6183
**6**	56.0098
**7**	53.4016
**8**	50.1384
**9**	55.6748
**10**	49.7106
**11**	54.6937
**12**	54.7161
**13**	47.8341
**14**	50.1740
**15**	55.8715
**16**	62.0570

**Table 3:** The affinity of selected compounds given as ChemPLP scores for LXRα (3IPS) compared to benzisoxazole and for LXRβ (1P8D) compared to 24(S),25-epoxycholesterol.

Receptor (Code) Compound	LXRα (3IPS)	LXRβ (1P8D)
Benzisoxazole	99.8329	
24(S),25-epoxycholesterol		96.3293
**1**	76.2498	90.3798
**2**	73.0104	85.7629
**3**	75.4276	87.6638
**4**	76.8951	88.1282
**5**	75.5349	87.5426
**6**	80.1341	94.7679
**7**	72.3868	94.1506
**8**	73.3147	94.8802
**9**	78.3528	96.6282
**10**	64.6718	90.4889
**11**	59.0236	82.4913
**12**	56.9901	73.6777
**13**	74.2402	89.7435
**14**	66.7384	77.9843
**15**	69.0156	78.0057
**16**	87.8329	88.7150

**Table 4:** ChemPLP scores for 16 observed compounds toward VEGFR2 (4ASD) in comparison to sorafenib (BAY 43-9006).

Receptor (Code) Compound	VEGFR2 (4ASD)
Sorafenib	107.7303
**1**	50.8489
**2**	47.5322
**3**	54.3167
**4**	48.4046
**5**	50.2772
**6**	49.4134
**7**	36.8851
**8**	37.1087
**9**	46.2666
**10**	40.0321
**11**	40.5274
**12**	32.1557
**13**	44.7827
**14**	31.6853
**15**	29.4592
**16**	47.5695

## Data Availability

The original contributions presented in this study are included in the article. Further inquiries can be directed to the corresponding author.
